# Recovery from coma of a patient having acute progression of primary central nervous system lymphoma using tirabrutinib and methylprednisolone

**DOI:** 10.1093/noajnl/vdaa164

**Published:** 2020-11-27

**Authors:** Takeshi Satow, Satoshi Horiguchi, Taro Komuro

**Affiliations:** Department of Neurosurgery, Nagahama City Hospital, Nagahama city, Shiga, Japan

**Keywords:** coma, corticosteroid, primary central nervous system lymphoma, progression, tirabrutinib

A patient with end-stage primary central nervous system lymphoma presented with acute deterioration of consciousness. MRI showed a diffuse high-intensity lesion involving the right thalamus and mesencephalon on FLAIR image. She had just begun to take tirabrutinib. Emergent high-dose intravenous methylprednisolone for three consecutive days was given, which resulted in complete recovery of consciousness. Tirabrunitib and maintenance dexamethasone was continued thereafter. Follow-up MRI disclosed improvement of diffuse abnormality at 1 month after this treatment. For patients with acute progression of recurrent CNS malignant lymphoma, tirabrutinib and corticosteroid could be one of the treatment options.

A 60-year-old woman was transferred urgently to our hospital due to her acute comatose state. She had been under treatment for primary central nervous system lymphoma (PCNSL) of diffuse large B cell type during the previous 2 years, including five courses chemotherapy using high dose of methotrexate, followed by whole brain irradiation for an intracranial metastasis at the right occipital lobe, 12 months after the initial presentation. About 5 months after that, dissemination to the fifth lumbar nerve was treated with focal irradiation. She had already been prescribed dexamethasone (0.5 mg/day).

A small focal lesion enhanced with gadolinium (Gd) in the right thalamus had already been observed at the time of lumbar irradiation therapy (17 months after the initial presentation), and this was followed up closely by neuroimaging. Two months later, MRI showed that the lesion had grown slightly, but she was still able to walk with assistance, and her KPS score was 70. One and a half months later, this homogenously enhanced lesion appeared to show a small ring enhancement, and the accompanying high intensity lesion had shrunk according to FLAIR imaging.

**Figure 1.**
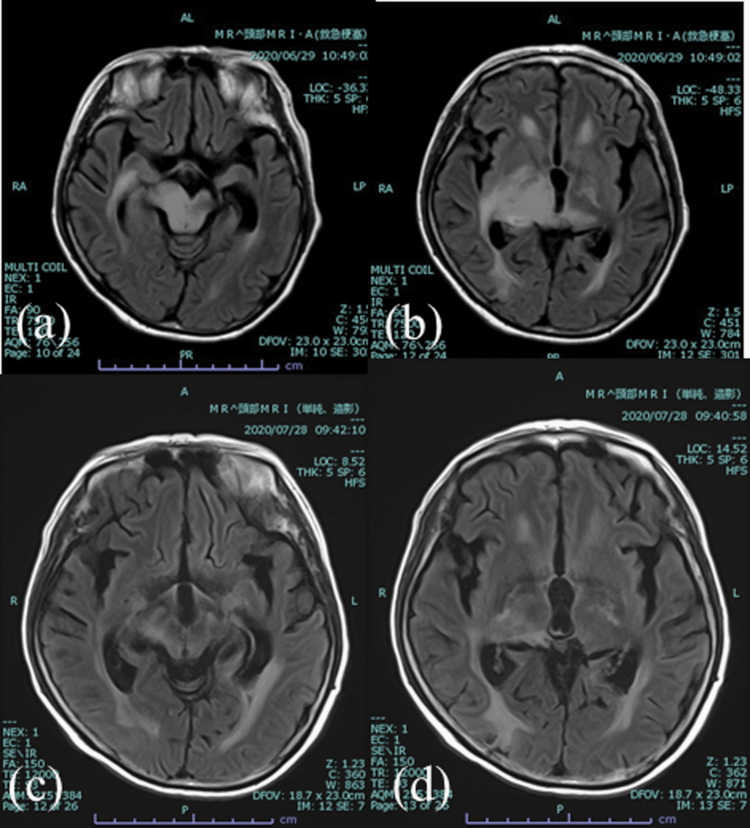
FLAIR image on admission (a,b) and 1 month after admission (c,d). The massive hyperintensity in the right thalamus and brainstem diminished significantly 1 month after treatment (c,d).

On admission, the woman was comatose (Glasgow Coma scale: 5) and had isocoric pupils with a sluggish reaction to the light reflex. Withdrawal response to pain stimuli was observed in all extremities, with no lateral asymmetry. MRI without Gd revealed a diffuse high-intensity lesion involving the right thalamus and mesencephalon on DW and FLAIR images (Figure 1a and 1b). She had just begun to take tirabrutinib (480 mg/day) as a second-line chemotherapy for the previous 2 days,^1^ because her daily living activities had deteriorated although her consciousness preserved. After admission, she was emergently given intravenous methylprednisolone (1000 mg/day) for three consecutive days, then a gradual decrease to the maintenance dose of dexamethasone (8 mg/day). Soon after the 3 days of methylprednisolone, she recovered consciousness to a state of full wakefulness. Motor weakness in all limbs persisted in practical voluntary movements, restricting her to a wheelchair. MRI disclosed that the hyperintensity lesion on DW and FLAIR images had diminished (Figure 1c and 1d), and the enhanced mass was stable at 1 month after admission. She was able to return home with a KPS of 30. She continued to take tirabrutinib for 1 month, and then discontinued it due to a skin rash. MRI taken 3 months after the treatment showed that the enhanced lesion in the right thalamus almost disappeared (data not shown).

In this present case, acute progression of recurrent CNS malignant lymphoma involving thalamo-mesencephalon was reversed, at least palliatively, via tirabrutinib and corticosteroid (methylprednisolone, dexamethasone). Acute deterioration might be a progression of the pre-existing recurrent lymphoma rather than radiation necrosis or leukoencephalopathy, given the neuroimaging features of the right thalamic lesion showing spontaneous remission followed by acute relapse. It is notably uncertain whether the reversal from apparently lethal brainstem damage is due to tirabrutinib or corticosteroid or both, based on this single case. As no treatment options exist other than corticosteroids in end-stage PCNSL, tirabrutinib might be an optional treatment to add.

Recently, the high efficacy of an irreversible selective inhibitor of Bruton’s tyrosine kinase (BTK), ibrutinib, on relapsed/refractory PCNSL has already been reported.^2^ Tirabrutinib (ONO/GS-4059, Ono Pharmaceutical) is a newly developed another drug for relapsed/refractory PCNSL, which highly selectively inhibits BTK irreversibly. Its safety, tolerability, and efficacy have only recently been reported.^1^ Further study is needed to learn more about real-world deployment of tirabrutinib for progressive stages of PCNSL.
